# Understanding multimorbidity requires sign-disease networks and higher-order interactions, a perspective

**DOI:** 10.3389/fsysb.2023.1155599

**Published:** 2023-06-06

**Authors:** Cillian Hourican, Geeske Peeters, René J.F. Melis, Sandra L. Wezeman, Thomas M. Gill, Marcel G.M. Olde Rikkert, Rick Quax

**Affiliations:** ^1^ Computational Science Lab, Institute of Informatics, University of Amsterdam, Amsterdam, Netherlands; ^2^ Department of Geriatric Medicine, Radboud University Medical Center, Nijmegen, Netherlands; ^3^ Radboudumc Alzheimer Centre, Radboud University Medical Centre, Nijmegen, Netherlands; ^4^ Department of Internal Medicine, Yale University School of Medicine, New Haven, CT, United States; ^5^ Institute for Advanced Study, Amsterdam, Netherlands

**Keywords:** multimorbidity, synergy, symptom-symptom interactions, Bayesian network, higher-order interactions, symptom-disease interactions, sign-sign interactions

## Abstract

**Background:** Count scores, disease clustering, and pairwise associations between diseases remain ubiquitous in multimorbidity research despite two major shortcomings: they yield no insight into plausible mechanisms underlying multimorbidity, and they ignore higher-order interactions such as effect modification.

**Objectives:** We argue that two components are currently missing but vital to develop novel multimorbidity metrics. Firstly, networks should be constructed which consists simultaneously of signs, symptoms, and diseases, since only then could they yield insight into plausible shared biological mechanisms underlying diseases. Secondly, learning pairwise associations is insufficient to fully characterize the correlations in a system. That is, synergistic (e.g., cooperative or antagonistic) effects are widespread in complex systems, where two or more elements combined give a larger or smaller effect than the sum of their individual effects. It can even occur that pairs of symptoms have no pairwise associations whatsoever, but in combination have a significant association. Therefore, higher-order interactions should be included in networks used to study multimorbidity, resulting in so-called hypergraphs.

**Methods:** We illustrate our argument using a synthetic Bayesian Network model of symptoms, signs and diseases, composed of pairwise and higher-order interactions. We simulate network interventions on both individual and population levels and compare the ground-truth outcomes with the predictions from pairwise associations.

**Conclusion:** We find that, when judged purely from the pairwise associations, interventions can have unexpected “side-effects” or the most opportune intervention could be missed. The hypergraph uncovers links missed in pairwise networks, giving a more complete overview of sign and disease associations.

## 1 Introduction

Multimorbidity, defined as the co-occurrence of two or more chronic diseases in the same individual, is highly prevalent in the general population, especially among older individuals (65% prevalence in persons aged 65–84 years and 82% in persons aged 85+) (Nguyen et al., 2019; Skou et al., 2022). With ageing populations, the number of people with multimorbidity is set to rise (Fortin et al., 2005; Salisbury et al., 2011; Barnett et al., 2012), leading to worse health outcomes and reduced quality of life (Huntley et al., 2012).

The single-disease paradigm is however still dominant in current clinical practices. In this approach, clinical symptoms and signs are linked to a single disease; and in case of multiple diseases, each disease is treated individually (Olde Rikkert et al., 2003). This approach is inappropriate for patients with multimorbidity for multiple reasons. Treating diseases in parallel often leads to a high total treatment burden and over-treatment, which can be exacerbated by treatment interactions (Boyd and Kent., 2014; Boyd and Kent., 2014). Treating each of a set of diseases individually, without considering the other diseases, is also inefficient and potentially harmful (Boyd and Kent, 2014).

Count scores and clustering methods characterize current multimorbidity research. However, these approaches yield no insight into plausible mechanisms underlying multimorbidity. The field of “network medicine” has brought the richer perspective of networks to the study of multimorbidity, with so-called “disease networks” to investigate the structure and dynamics of complex patterns in multimorbidity and “disease trajectories” of patients (Barabási et al., 2010; Jones et al., 2022). However, disease networks are currently defined using (pairwise) associations between diseases, which miss possible higher-order (synergistic) associations. Existing works which use hypergraphs do not use them to represent non-linear multivariate associations but, e.g., to count co-occurrences of diseases (Haug et al., 2021; Rafferty et al., 2021).

Our first argument is that symptoms, signs, and disease states should be part of one and the same network. In contrast, the single disease burden has so far been dominant in practice, so the symptoms and signs were not the primary focus in the past. Tripp-Reimer et al. (2020) allude to symptoms not being widely used due to their subjectivity and difficulty to access from health records. We believe it is nevertheless important to combine them in one network because symptoms and signs form plausible pathways between diseases. The definitions of symptoms, signs, and diseases is a long-standing debate with no consensus yet (Olde Rikkert et al., 2022). Throughout the article we use the term *sign* to refer to a an objective indication of some medical fact, *symptom* to a subjective experience of a patient, and *disease* to the diagnosis of disease states.

Our second argument is that we must go beyond pairwise interactions by including also so-called “synergistic associations” in the network models. Synergistic interactions are often referred to as effect modification or mediation, and traditionally captured by including interaction terms in regression models (more specifically: polynomial series). There are two shortcomings with this approach. The first is that it is parametric: a very specific choice of functional form must be chosen (usually a multiplication of variables), which can be suitable for capturing certain non-linear effects (e.g., an AND-gate for multiplication) but unsuitable for other non-linear effects (e.g., no sequence of AND-gates can represent an XOR-gate) (Liang and Han, 2012). The second shortcoming is that, depending on the choice of functional form, many terms may be needed to represent a given non-linear association. Making matters worse, for a single choice of functional form, multiple different loadings of the interaction terms may lead to the same prediction power. This is referred to as the multiplicity problem (Tsuchiya, 2014; Marx et al., 2020). This makes the interpretation of the interaction terms, representing the synergistic interaction, difficult and ambiguous. Therefore, we use a non-parametric approach, based on Shannon’s information theory. More precisely we will use the O-information heuristic because of its computational efficiency (Rosas et al., 2019).

We first demonstrate our two arguments in a synthetic Bayesian network (BN) model constructed using both pairwise and synergistic associations, which we use to generate synthetic datasets with 100,00 realisations. In healthcare, BNs are used in aiding decision-making processes for diagnosis, prognosis, and treatment selection (Lucas et al., 2004). Binary nodes of diseases, disorders and risk factors, along with background information, have been used in BNs to study the multimorbidity rate, and co-occurrences of multiple diseases (Lappenschaar et al., 2013b; Lappenschaar et al., 2013a; Song et al., 2022). However, to our knowledge, there does not appear to be BNs studying multimorbidity using symptoms, signs and diseases in one network, even though several works mention the need to combine these elements (Willadsen et al., 2016; Yarnall et al., 2017; Griffith et al., 2018). Then we use *EASYcare Twostep Older persons Screening data* (van Kempen et al., 2015) to identify synergistic associations and highlight their prevalence. The code and generated data are provided at https://github.com/CillianHourican/Synergistic-Networks.

## 2 Methods

### 2.1 A primer on synergistic interactions

Synergistic effects are common in complex systems and can take many forms. The clearest evidence of a synergistic effect is when independent random variables individually cannot predict a target variable (zero correlation) but when considered together they can (non-zero correlation). This is not the only situation where synergistic effects take place: synergy is also present when the sum of the pairwise correlations between the independent variables and the target variable is less than the multivariate correlation of the independent variables with the target variable. Throughout the article, “correlation” is taken to be measured using Shannon’s mutual information (Cover and Thomas, 2005). This is non-parametric and captures both linear and non-linear associations (Kinney and Atwal, 2014).

A classic example of a purely synergistic interaction is when predicting the output Y of an XOR-gate with two binary inputs $S_{1},S_{2}$ . An XOR gate effectively indicates whether the inputs are equal (output 0) or unequal (output 1). Intuitively this is clearly a type of relationship where the output cannot be predicted from one input alone. To see this, let an XOR gate output Y=1 whenever exactly one input equals 1 and the other 0, and Y = 0 otherwise. Furthermore, let each input be 50/50 distributed. Without observing any input values, the target Y has a marginal probability distribution which is maximally uncertain (also 50/50 distributed):

$$P\left(y=1\right)=P\left(y=0\right)=0.5$$

This distribution does not change if we condition on either one of the input variables:

$$P\left(y=0|S_{1}\right)=0.5=P\left(y=1|S_{1}\right)$$

$$P\left(y=0|S_{2}\right)=0.5=P\left(y=1|S_{2}\right)$$

In words, observing either input alone does not improve our information (ability to predict) the output. Indeed, using mutual information we see that separately each input tells us zero information about the response:

$$MI\left(S_{1}:Y\right)=0,MI\left(S_{2}:Y\right)=0$$

In contrast, the joint random variables specify all the information about the target: $MI\left(S_{1},S_{2}:Y\right)=1.$

This is because observing both inputs fully determines the output Y (100/0 or 0/100 distributed). Concluding, the information about the target in an XOR-gate is not stored in either single input (pairwise correlation) but is stored completely synergistically in the combination of both inputs.

This is important because many networks are constructed using pairwise correlations. In our example, though, the pairs $\left(S_{1}Y\right),\left(S_{2},Y\right)$ have zero correlation. No algorithm that depends on pairwise correlations can find the synergistic relationship. This is the essence of our argument against focusing purely on pairwise association networks and for explicitly including synergistic interactions. This argument remains true when building correlation networks based on conditioned correlations or conditional independences (James et al., 2016).

Quantifying the exact amount of synergy between variables remains an open problem, with frameworks such as partial information decomposition aiming to decompose information between variables into unique, redundant, and synergistic information (Timme et al., 2014; Grith, 2014; Olbrich et al., 2015; Finn and Lizier, 2018; Lizier et al., 2018; Procaccia et al., 2023). In our current study, we use the *O-information* heuristic when analysing data to assess if synergy is indeed present (Rosas et al., 2019). This heuristic is easy to compute but is conservative, i.e., some interactions may be at least partly synergistic while the O-information measure fails to detect them (Rosas et al., 2019). The converse is not possible: if O-information infers a synergistic relationship then there must be significant synergy. This metric has been utilized in neuroscience, in fMRI signals to characterise higher-order communication between different regions of the brain, and to capture neural spiking dynamics (Stramaglia et al., 2021; Santos et al., 2023). This information-theoretic approach captures synergy by computing (multivariate) mutual information and is model free.

However, capturing synergistic interactions is relevant in many domains, such as synergistic drug-drug combinations, increased co-occurrances of diseases, and functional activity in the brain (Tallarida, 2011; Timme et al., 2014; Wildenhain et al., 2015; Rønneberg et al., 2021). As prediction models become ever more complex, feature attribution methods have become essential tools to provide explainability and interpretability. This often involves performing a sensitivity analysis or breaking down a complex system into its individual components and analysing how these components interact to produce the overall behaviour of the system. This process allows us to better understand the underlying mechanisms and processes in the model. Approaches such as Integrated gradients, Layer-wise Relevance propagation and Gradient-weighted Class Activation Mapping have been developed for neural networks, while Sobol’ Indices and Shapley values are used for a much broader range of models (Owen, 2014; Selvaraju et al., 2017; Montavon et al., 2019; Gevaert and Saeys, 2022; Lundstrom et al., 2022). These approaches require two choices to be made; what model should be fit to the data and what attribution technique should be utilised. This contracts with the information-theoretic approach which is directly applied to data. Further discussion on different approaches to capture synergy, O-information and mutual information variants is provided in the Supplementary Material.

### 2.2 Creating a synthetic network model with synergistic interactions

**FIGURE 1:**
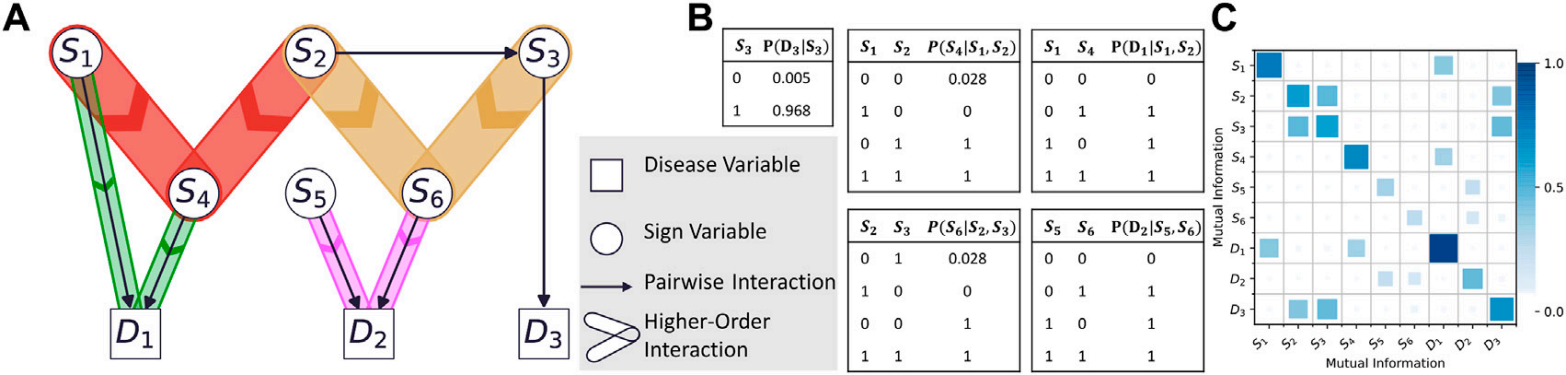
**(A)** The synthetic network model with pairwise and synergistic interactions. The narrower hyperedges indicate interactions less synergistic than the wider hyperedges. **(B)** The conditional probability table with variables $S_{1},S_{2},P\left(S_{4}|S_{1},S_{2}\right)$ can be interpreted as follows; The first row shows a 2.8% chance of $S_{4}$ being on when $S_{1}$ and $S_{2}$ are off. If $S_{1}$ is on and $S_{2}$ is off, $S_{4}$ is always off. If $S_{1}$ is off and $S_{2}$ is on, $S_{4}$ is always on. And if both $S_{1}$ and $S_{2}$ are on, $S_{4}$ is always on. **(C)** Mutual information scores for each pair of variables from a synthetic dataset, with 100,000 realisations, generated by the model. Larger and darker squares show stronger pairwise associations.

Our synthetic model (Figure 1A) is a BN model of sign and disease variables. In this example model we do not differentiate between symptoms and signs and refer to them simply as signs. BNs are causal graphs which are directed and acyclic and its edges are defined by conditional distributions among stochastic variables, which are shown as nodes (Briganti et al., 2022).

Our running example follows Lucas et al. (2004) and Borsboom (2017) where signs can cause (the diagnosis of) diseass, but diseases do not directly cause sign activation. In other words, while signs are causally linked and can have both incoming and outgoing connections, diseases are inferences derived from (a combination of) signs. Notwithstanding, others do consider causal relations from diseases to signs (Richens et al., 2020). It is important to note, however, that our main conclusions would remain unchanged if adding such causal relations.

Our synthetic network has three independent nodes $\left(S_{1},S_{2},S_{5}\right)$ , without incoming links, each with a Dirichlet prior distribution (Koller and Friedman, 2009). For further simplicity, our model has only binary nodes (“on/off”) which is sufficient for our demonstration, but the described approach is valid for both discrete and continuous data. Node $S_{3}$ has a large mutual information with its parent node $S_{2}$ , as represented by the direct link from $S_{2}$ to $S_{3}$ . Similarly for the edge ${S_{3}\to D}_{3}$ . Highly synergistic random variables from Quax et al. (2017) are appended to the system, as shown by the wide edges for $\left(S_{1},S_{2}\right)\to S_{4}$ and $\left(S_{2},S_{3}\right)\to S_{6}$ , which we refer to as triplets. Arrows on each edge indicate the causal direction in the network. We do this by joint optimization of conditional probabilities by maximizing the so-called Whole-Minus-Sum objective function, where we aim to minimise the pairwise mutual information and maximise the combined mutual information of both inputs about the target. For the edge $\left(S_{1},S_{2}\right)\to S_{4}$ , this synergistic association ensures the mutual information of pairs $MI\left(S_{1},S_{4}\right)$ and $MI\left(S_{2},S_{4}\right)$ are approximately zero, meaning no pairwise association, while there is a large mutual information when jointly considering both nodes on the target. A similar setup is seen with the triplet $\left(S_{2},S_{3}\right)\to S_{6}$ . However, in this case the mutual information $MI\left(S_{2},S_{3}\right)$ is significant, as shown by the directed edge, thus making the synergistic effect weaker. Finally, OR gates are used for the dynamics of diseases $D_{1}$ and $D_{2}$ , where $D_{1}$ activates if either $S_{1}$ or $S_{4}$ activates, while $D_{2}$ activates if $S_{5}$ or $S_{6}$ activates. This combination of pairwise and synergistic edges results in a hypernetwork structure.

We assume this ground truth is known, with the focus on illustrating how synergistic interactions influence model interventions. We do not focus on techniques to reconstruct such a BN from data, or the presence of latent variables in this synthetic model. The only latent variables present are noise-generating functions. Pairwise associations alone cannot reconstruct this network structure. Figure 1B shows a matrix of mutual information values for each pair of variables, computed from a synthetic dataset generated by the model. Each shaded square represents an edge in the corresponding pairwise network. However, some associations are then missing, which incorrectly results in three disconnected pairwise network forming (See Supplementary Material). Including synergistic associations is needed to form the complete network structure, resulting in a hypernetwork structure.

### 2.3 Model interventions

Next we perform two types of interventions to gain insight into this complex system. We first simulate an intervention at the population-level by picking a sign variable and nudging its marginal distribution slightly, acting as a mass influence on the whole population. The marginal probability distribution reflects the prevalence of the sign in the population. By nudging we mean moving probability mass from one state to the other in the probability mass function (pmf) for a variable, slightly changing its marginal probabilities while keeping all conditional probabilities unchanged, in causal inference this is known as a soft intervention (Eberhardt and Scheines, 2007). We remind the reader that in our model, conditional probabilities reflect causal mechanisms (e.g., smoking leads to increased risk of lung cancer), so we only change marginal probabilities. This type of population-level intervention is akin to a shift in society’s normal behaviour or trying to manipulate population-level causes of the incidence sickness through public health measures rather than protecting high risk individuals within a population (Rose, 2001). For example, a nudge of 0.1 on node S may correspond to increasing $P\left(S=1\right)=0.3$ to $\hat{P}\left(S=1\right)=0.4$ , which changes the probability by $\left|P-\hat{P}\right|=0.1.$ The effect of the intervention is then computed using the change in log-odds ratio for each node in the network. In the binary setting, a nudge is not probabilistic and so is fully reproducible. However, when using multivariate or continuous variables there are many ways to shift the PMF, and some sampling techniques would be needed to analyse the effects of nudge interventions, as the results would vary each time.

Our second simulated intervention, on the other hand, pertains to clinical practice where we are concerned with a single patient rather than population-level scores. Here we may ask: “Given that this patient has this sign, what other sign is she likely to acquire?” Or, “Can the onset of a particular sign be prevented by a strategic intervention, given the other signs of a patient?” In this case it no longer makes sense to speak of a pmf of a sign: a given patient has a well-defined value for a sign, which in our binary formalism means a patient either has or does not have a particular sign. To this end, individual-level interventions were performed by generating one realisation (value for each sign and disease in the network), changing the state of one or more nodes, and recording the downstream changes. This is akin to hard interventions in do-calculus, as we are forcing a node to take a certain value (Eberhardt and Scheines, 2007).

### 2.4 Uncovering synergistic associations in medical data

The prevalence of synergistic associations was illustrated using the *O-information* heuristic. The data set used is the *EASYcare Twostep Older persons Screening data* (van Kempen et al., 2015) This is a group of 587 older persons aged 60+ from GP practices in the Netherlands, and holds cross-sectional data on 37 signs along with binarized *Cumulative Illness Rating Scale-Geriatric* (CIRS-G) data on the absence or presence of diseases (operationalized as each of the 14 CIRSG-subscales being equal to or greater than 2) taking from a complete medical examination. A permutation test was used to determine the significance of each synergistic triplet using the Benjamini-Hochberg procedure (BHR) with a family-wise error rate of 0.15 (Ferreira and Zwinderman, 2006).

## 3 Results

### 3.1 Interventions at the population-level

**FIGURE 2:**
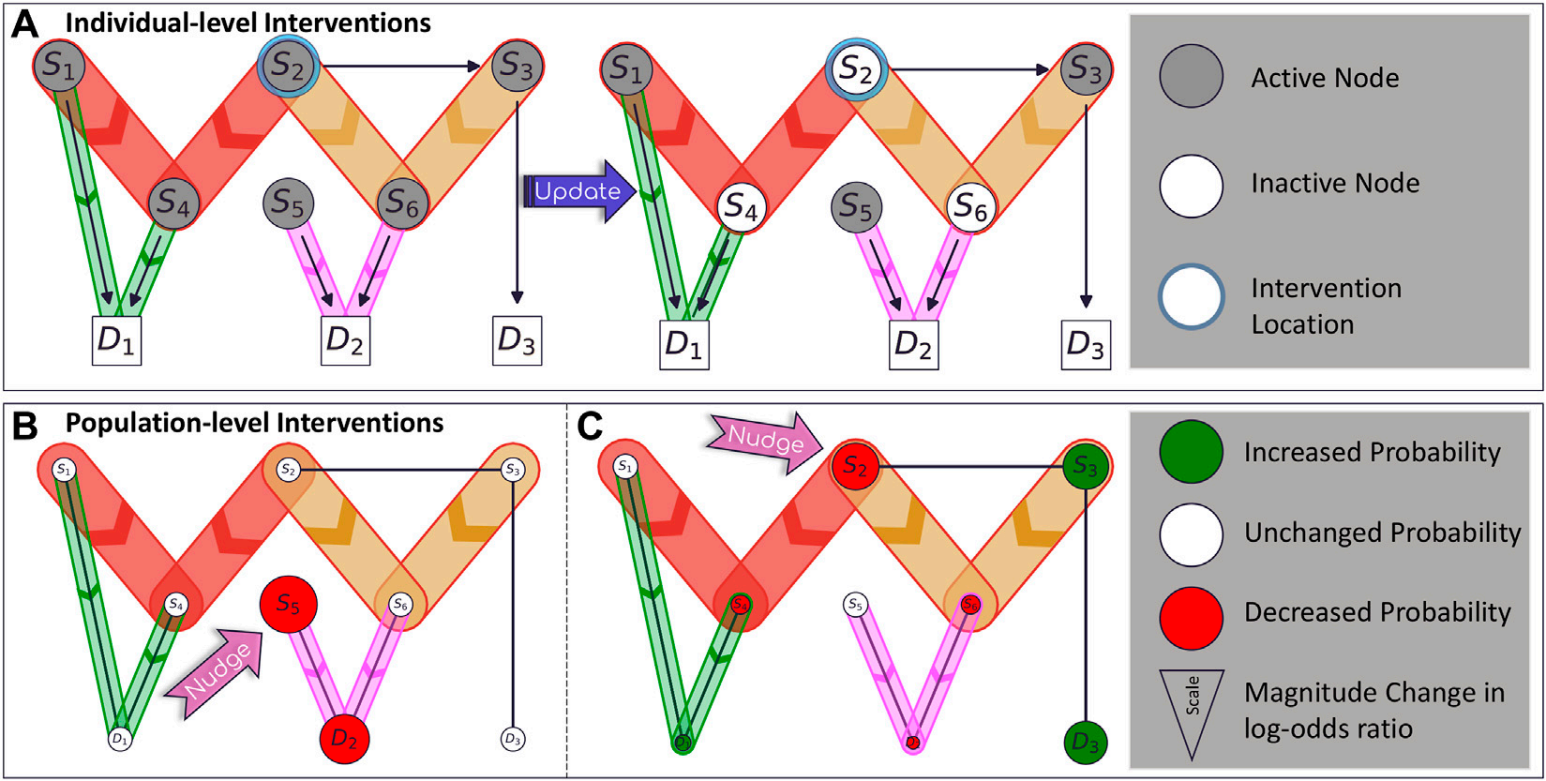
**(A)** An example realisation of the network. Coloured nodes are active (“on”) in the “patient” while white nodes are inactive. An individual-level intervention turns off one node, updating downstream nodes as they also become inactive. **(B)** The effect of a (population-level) nudge intervention on node $S_{5}\;.$ This intervention updates downstream marginal probabilities from where the nudge intervention occurred. Larger nodes indicate a larger change in the log-odds ratio compared to the system state before interventions. Green nodes show increased prevalence (marginal probabilities), red show decreases, and white shows no change. **(C)** The corresponding effect of nudging node $S_{2}$ .

First, we nudge the pmf of $S_{5}$ by 0.2 (Figure 2B), changing its marginal probabilities while keeping all conditional probabilities unchanged. This carries through to a slight increase in the prevalence of $D_{2}$ , while all other variables are unaffected. This direct causal effect is expected by the pairwise network, since a pairwise connection is inferred in the network from $S_{5}$ to $D_{2}$ . By construction, no other nodes are descendants of this node, and so are not affected.

In a second intervention, we nudge the prevalence of $S_{2}$ (Figure 2C). This intervention filters through the network, changing the marginal probability (prevalence) of several other nodes in the network. The direct pairwise links $\left(S_{2}\to \;S_{3}\right)$ and then $\left(S_{3}\to D_{2}\right)$ catered for these secondary effects. However, by construction, the synergistic associations also facilitate (minor) changes in distributions of $S_{4}$ and $S_{6}$ , which subsequently caused changes in $D_{1}$ and $D_{2}.$ The pairwise network misses these changes to $S_{4},S_{6},D_{1},D_{2}$ .

### 3.2 Interventions at the individual-level

Suppose a patient presents with signs ${S_{1},S}_{2},S_{3},S_{4},S_{5},S_{6}$ (Figure 1C), where we have no reason to prioritise treating one sign over another. When considering only the direct pairwise associations, we incorrectly conclude that the active signs are not connected to each other. This implies that each sign must be treated individually, meaning four separate treatments for the patient. However, when synergistic associations are included, we see that not all interventions are necessary. Sign $S_{2}$ has a synergistic causal effect on sign $\left(S_{4},S_{6}\right)$ , so this intervention will impact both signs, as making $S_{2}$ inactive also makes both $\left(S_{4}{,S}_{6}\right)$ inactive. Ignoring synergistic interactions leads to overtreatment of the patient, as this involves intervening on every active sign node.

### 3.3 Synergistic associations in medical data

For computational efficiency, we first found triangles of low dyadic mutual information (MI < 0.05) as these are likely to be missed when performing a pairwise analysis. Of these, 184 triplets were (Quax et al., 2017) significantly synergistic, with *p*-values less than 0.05 using the Benjamini-Hochberg algorithm to account for the false discovery rate.

One such example is weight loss $\left(w\right)$ , dyspnoea $\left(d\right)$ , and locomotor pain $\left(pl\right)$ , which all have weak dyadic associations but significant synergistic associations. A patient with only joint pain may be advised to lose weight to reduce the strain on their joints (King et al., 2013). Similarly, a patient with dyspnoea may be advised to lose wight to alleviate its effects (Bernhardt and Babb, 2014; Bernhardt et al., 2019). However, if the patient has both joint pain dyspnoea, their intensity and duration of physical activity is further limited. This comorbidity further increases the patients suffering, making it much less likely that losing weight to reduce joint pain succeeds (Hayen et al., 2013). Here the probability of weight loss (0.097) remains (almost) unchanged given the presence/absence of one sign:

$$P\left(w|pl=1\right)=0.094$$

$$P\left(w|d=1\right)=0.084$$

$$P\left(w|pl=0\right)=0.077$$

$$P\left(w|d=0\right)=0.088$$

However, conditioning on both variables results in a much larger change of probabilities;

$$P\left(w|pl=0,d=0\right)=0.048$$

$$P\left(w|pl=0,d=1\right)=0.169$$

$$P\left(w|pl=1,d=0\right)=0.126$$

$$P\left(w|pl=1,d=1\right)=0.045$$

Other synergistic triplets include (*Stiff restricted, UTI, CIRS-G Lower GI*) and (*Movement limitation, Hearing problem, CIRS-G Heart*). A collection of synergistic triplets is presented in the Supplementary Material.

## 4 Discussion

Multimorbidity research is moving from individual treatment of diseases toward a complex system view of multimorbidity, but it remains unclear how exactly this is to be achieved. Network models of diseases and disease clusters, which are based on dyadic associations, are becoming increasingly popular to study multimorbidity (Barabási et al., 2010; Jones et al., 2022). However, we have shown that such networks may miss important synergistic relationships in data, so we argue that polyadic associations be included in such networks. The prevalence of synergistic associations was demonstrated using a medical dataset where we found 184 triplets to be significantly synergistic. Although more datasets should be analysed to uncover the true prevalence of synergistic associations, this result at least demonstrates that further investigation is warranted.

Melis et al. (2017) argue that methods modelling multimorbidity at the disease level may have limited value, as they do not accurately inform clinical decision making. With increasing disease burden, the presenting signs become less specific, and it is more difficult to identify a clear set of underlying diseases. Therefore, network models must uncover relationships among signs and diseases. Constructing such networks with synergistic associations permits interdependencies among signs and diseases to be uncovered while capturing associations that may be missed when only considering pairwise associations.

There are three key points we wish to take from the analysis of our synthetic model. Firstly, synergistic associations may cater for changes in the system that cannot occur from pairwise associations, and thus statistical analyses should not just explore pairwise correlations but also involve additional analyses that are able to uncover these synergistic association in empirical data to fully determine intervention effects.

Secondly, local interventions in complex systems can have global effects. When considering interventions, we must look beyond variables directly associated with our variable of interest and consider how modifying a single node changes the whole system. Networks built from pairwise associations uncover links that may overlook synergistic interactions, and so may not capture the true global effects.

Thirdly, networks constructed from pairwise associations uncover links that may overlook synergistic interactions. This may lead to unexpected outcomes because we overlook links that do exist. If we only try to construct the causal structure using pairwise associations, we may miss the carry-through effects via synergistic interactions, expect inaccurate global-effects, and intervene on more or less nodes than needed.

However, these approaches are not without limitations. Finding synergistic associations brings additional computational costs, especially when including higher-order groups, such as quadruplets and quintuplets. Capturing higher-order associations is constrained by the availability of data, which limits the complexity of the resulting hypergraph. Quantifying the amount of synergy also remains a theoretical problem (Grith, n.d.; Quax et al., 2017; Finn and Lizier, 2020). Spurious findings should also be considered when constructing network models (Fried, 2017; Epskamp and Fried, 2018). Additionally, integrating these associations into prediction and causal models remains an open problem. Nevertheless, it is important to be aware of such synergistic associations, especially when they uncover relations missed in the pairwise-based network.

To conclude, patients with multimorbidity have complex patterns of signs and diseases, each of which should simultaneously be included in networks constructed to study multimorbidity. Studying pairwise associations alone is not sufficient in such a complex setting. Synergistic associations between signs occur and must be included in future analysis. Our arguments were supported by both results from the simulations in the synthetic model and analyses of real-world data.

## Data Availability

The datasets presented in this study can be found in online repositories. The names of the repository/repositories and accession number(s) can be found below: https://github.com/CillianHourican/Synergistic-Networks.
