# Supplementary material for: Understanding multimorbidity requires sign-disease networks and higher-order interactions, a perspective
Source: Front Syst Biol. 2023 Jun 6;3:1155599. doi: 10.3389/fsysb.2023.1155599 (PMC10557993; doi:10.3389/fsysb.2023.1155599)
Supplement: Supplementary file 1 [file Table1.pdf]

Supplementary Material

Understanding multimorbidity requires sign-disease networks and higher-order interactions, a perspective.

Cillian Hourican, Geeske Peeters, René Melis, Thomas M. Gill, Sandra Wezeman, Marcel Olde Rikkert, Rick Quax

*** Correspondence:** Cillian Hourican: c.j.hourican@uva.nl

1 Intuitive examples of synergistic triplets and XOR gates

Suppose we have two binary variables. An XOR gate is a logical gate which returns the value one when both inputs are different, and zero when both inputs are the same. The table below shows an XOR gate. Let's assume the third column is what we want to predict., and columns one and two are independent, each with a probability of 0.5.

Supplementary Table 1 An example of an XOR gate.

Switch 1	Switch 2	Lamp
**Off**	Off	Off
**Off**	On	On
**On**	Off	On
**On**	On	Off

We know probability the lamp is on = 0.5. If we learn that switch 1 is on, this tells us no information about the lamp being on/off. Alternatively, if we know the status of switch 2 but not switch 1, we gain no information about the lamp being on/off. We only get more information about the lamp if the state of both switch 1 and switch 2 are known. Here we would say the information about the lamp is not contained in either switch (i.e., no pairwise/dyadic mutual information), but is contained synergistically in their combination.

Supplementary Table 2 An example of a relationship with both pairwise and synergistic associations.

Sex	Intercourse	Pregnant
Male	No	No
Male	Yes	No
Female	No	No
Female	Yes	Yes

In the second table we want to get more information about a person being pregnant. We know this has a 25% probability. However, once we know the sex the pregnant probability changes to 0% or 50% for male/female. So, learning this input in isolation gives us information about being pregnant. Similarly for knowing intercourse but not the sex. Here we would say there is pairwise (dyadic) mutual information between each input and the output. Although indeed we need to know both sex and intercourse to know if the person is pregnant, so there is some synergistic information contained in their combination.

Using synthetic data, we find that the second table has an O-information score of ≈ -0.19. The negative value indicates that more information is contained synergistically between the two variables than through the pairwise associations. The synergistic association can also be seen where the sum of pairwise mutual information values $MI(sex, pregnant) \approx 0.3 \approx MI(Intercourse, pregnant)$ is less than the mutual information of the whole: $MI([sex, intercourse], pregnant) \approx 0.81$.

The first table is fully synergistic, while the second is mixed with both pairwise and higher-order associations. However, both contain synergistic information, and these are what we try to find in the data. The metric I'm currently using (O-information) looks at the balance between these pairwise and higher-order associations. When more information is contained synergistically than through pairwise associations, then it classifies the triplet as synergistic.

2 Mutual Information variants and O-information

The mutual information between two random variables X and Y is a measure of the amount of information that one variable has about the other. This pairwise measure ignores any other variables that may be present, and can be expressed as:

$$MI(X;Y) = H(X) - H(X|Y) = H(Y) - H(Y|X) = H(X) + H(Y) - H(X,Y)$$

where $H(X)$ and $H(Y)$ are the entropy of X and Y, respectively, and $H(X|Y)$ and $H(Y|X)$ are the conditional entropies of X given Y and Y given X, respectively. The mutual information between two variables X_1 and X_2, about a third variable Y, written as $MI(X_1, X_2; Y)$, can be computed by using the

composite of X_1 and X_2 as a multivariate variable. The equation is found by replacing X by (X_1, X_2) in the equation above.

Conditional mutual information is the information two random variables X, Y share, given the knowledge of a third random variable Z, which can be written as

$$MI(X;Y|Z) = H(X|Z) - H(X|Y,Z),$$

Where $H(X|Z)$ is the conditional entropy of X given Z, which measures the uncertainty of X given knowledge of Z and $H(X|Y,Z)$ is the conditional entropy of X given both Y and Z, which measures the uncertainty of X given knowledge of both Y and Z. Intuitively, this measures how much two variables are related, while taking into account the influence of a third variable.

The Total Correlation (TC) and Dual Total Correlation (DTC) are two common multivariate extensions of mutual information, which can be written as

$$\text{TC}\,(X_1, \ldots, X_n) := I(X_1; X_2) + I(\boldsymbol{X}_1^2; X_3) + \cdots + I(\boldsymbol{X}_1^{n-1}; X_n)$$
$$\text{DTC}\,(X_1, \ldots, X_n) := I(X_1; X_2 \mid \boldsymbol{X}_3^n) + I(\boldsymbol{X}_1^2; X_3 \mid \boldsymbol{X}_4^n) + \cdots + I(\boldsymbol{X}_1^{n-1}; X_n)$$

where $\boldsymbol{X}_i^j = (X_i, .., X_j)$ is a shorthand notation. Both quantities are equal to the mutual information for two variables. TC and DTC are zero if, and only if, the random variables are independent. TC accounts for collective constraints, while DTC measures the shared randomness (Rosas et al., 2019). The O-information is then defined by their difference

$$\Omega_n(X_1, \ldots, X_n) = \text{TC}\,(X_1, \ldots, X_n) - \text{DTC}\,(X_1, \ldots, X_n),$$

where $\Omega_n(X_1, \ldots, X_n) < 0$ implies a dominance of higher-order constraints in the data, which we refer to as synergy, and $\Omega_n(X_1, \ldots, X_n) > 0$ implies a dominance of low-order constraints, which implies redundant information. A more complete introduction can be found in (Marinazzo et al., 2022; Rosas et al., 2020).

3 An Example of synergy missed by O-information

The O-information identifies the primary trend of information in data and classifies it as either synergistic or redundant. However, in cases where both trends coexist, they may negate each other, causing some synergy to go undetected. For instance, suppose we have two independent random variables $X_1 \sim Binomial(0.5)$, $X_2 \sim Binomial(0.5)$ along with the output variable $Y = Y_1 + Y_2$ where $Y_1 = XOR(X_1, X_2)$ and $Y_2 = X$. Here, X_1, X_2, and Y form a triplet for which we calculate the O-info, which gives a score of zero. It is not dominated by either synergistic or redundant information. However, the variable Y is composed of synergistic information from the XOR-gate and redundant information from the copy of X_1.

4 Partial Information Decomposition

Partial information decomposition (PID) is a framework in information theory that aims to decompose mutual information between variables into unique, redundant, shared and synergistic information. Although many approaches exist to decompose the mutual information, no measure has been found that satisfies all three axioms "that any reasonable measure for shared information [$I_\cap$] should fulfil" (Olbrich et al., 2015) -symmetry, self-redundancy and monotonicity - for an arbitrary

number of source variables to a target. Finn (2018) write the axioms, for a target variable T and elements of the set of all predictors (sources) $A_1, \dots, A_k$, as

Axiom 1 (Symmetry). Redundant information is invariant under any permutation σ of sources

$$I_\cap(A_1, \dots, A_k; T) = I_\cap(\sigma(A_1), \dots, \sigma(A_k); T).$$

Axiom 2 (Monotonicity). Redundant information decreases monotonically as more sources are included,

$$I_\cap(A_1, \dots, A_{k-1}; T) \geq I_\cap(A_1, \dots, A_k; T)$$

with equality if $A_k \supseteq A_i$ for any $A_k \supseteq A_i$ for any $A_i \in \{A_1, \dots, A_{k-1}\}$.

Axiom 3 (self-redundancy). Redundant information for a single source A_i equals the mutual information, $I_\cap(A_i; T) = I(A_i; T)$.

5 Synergy Constructs in other domains

Capturing synergistic interactions is relevant in many domains, where the definition of synergy also varies, such as synergistic drug-drug combinations, increased co-occurrances of diseases, and functional activity in the brain (Tallarida, 2011; Timme et al., 2014; Wildenhain et al., 2015; Rønneberg et al., 2021). Also in AI and machine learning, techniques such as Shapley values exist to quantify feature importance and access synergies (Procaccia et al., n.d.)

While studying multimorbidity via disease occurrences, Lappenschaar (2013b) considers synergy as a significant deviation in the cumulative incidence rates of disease combinations resulting from increased occurrence, compared to what would be expected based on individual cumulative incidence rates, assuming statistical independence.

The search for synergistic interactions between drugs is also an active area of research. In these cases, the focus is on synergistic effects/responses rather than synergistic associations/information. They rely on the difference in the resulting effects is computed when drug does combinations are varied. In (Tallarida, 2011; Rønneberg et al., 2021) synergy is defined when the response curve from the different drug combinations differs significantly from the expected curve. However, this is limited by the choice of functional form of the expected response curve, be it linear, a specified non-linear function or a Gaussian Process. Furthermore, it is not possible to apply such an analysis on the multimorbidity level when comparing symptoms, signs, and diseases as, while drug doses can be altered on a continuous scale, often the occurrences of symptoms/signs are binary or discrete. Wildenhain (2015) also aims to predict synergistic effects of drug combinations on a target using machine learning methods.

Comparing Shapley values is another technique in AI and machine learning for explaining model predictions by quantifying the contribution of each feature to the final outcome. By measuring the marginal contribution of each variable, Shapley values can reveal how variables interact and combine to produce an overall effect. They can also be used to measure the extent to which two or more variables work together to produce a certain outcome, which can be helpful in identifying synergistic associations. (Procaccia et al., n.d.)

In our current study, we use the O-information heuristic when analysing data to assess if synergy is indeed present (Rosas et al., 2019). This heuristic is easy to compute but is conservative, i.e., some interactions may be at least partly synergistic while the O-information measure fails to detect them (Rosas et al., 2019). The converse is not possible: if O-information infers a synergistic relationship then there must be significant synergy. This metric has been utilized in neuroscience, in fMRI signals to characterise higher-order communication between different regions of the brain, and to capture neural spiking dynamics (Santos et al., n.d.; Stramaglia et al., 2021). This information-theoretic metric can be directly applied to data (continuous or discrete) without the need to construct and validate a prediction model.

6 The sufficiency of binary variables

The metrics used in this paper to infer synergy rely on the computation of mutual information. Binary data is sufficient for such computations because mutual information can quantify the degree of statistical dependence between two discrete variables, and binary variables allow for easy calculation of the necessary joint probabilities. This is illustrated with the examples of XOR-gates and synergistic variables appended in the network using SRVs.

Continuous data is not as straightforward to analyse as we often need data to be discretized when computing joint probabilities to compute mutual information. Furthermore, the number and size of intervals into which continuous data are discretized can affect the results obtained. This may potentially lose some information.

As mentioned in the main text, most existing data sources only report the presence of a symptom/sign, but not severity. While for some symptoms a continuous measure may be possible (e.g pain) for others it is not (e.g a fall occurs). The limitations and complexity of computing synergy from continuous variables is not addressed in the paper as it is likely not relevant for datasets collected to study multimorbidity through symptoms and signs.

7 Reconstructing the synthetic network structure using pairwise associations

Using synthetic data from our model, we can explore how well we can reconstruct the association networks by computing pairwise and synergistic associations. For pairwise associations, one approach is to compute partial correlations. The figure above shows the network of all non-zero partial correlations. This network has many spurious, and non-significant associations. To remove these edges, we can set a threshold for correlation strengths or use regularisation. Supplementary Figure 1 shows the corresponding network when only correlations with magnitude of at least 0.2 are included. This results in three disconnected pairwise networks forming. To fully characterise all correlations in the data, synergistic associations must also be included.

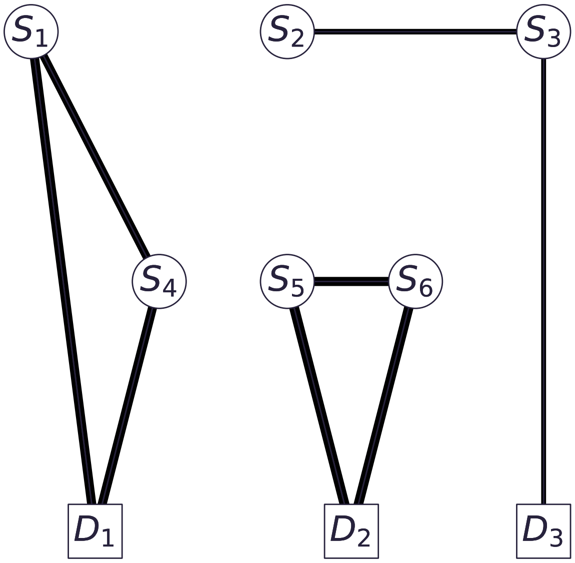

Supplementary Figure 1 Showing a network constructed from the synthetic network data, using pairwise associations. This incorrectly results in three disjoint networks.

8 O-information triplets from the synthetic model

To determine the significance of each synergistic triplet we ran a permutation test on all 84 triplets. Of these, 30 were found to be significantly synergistic, with p-values less than 0.05. The problem of multiple comparisons was not accounted for as this is just for illustration purposes. The top-ranking synergistic triplets, with O-info scores below -0.07 are shown in the table below.

Supplementary Table 3 Synergistic triplets from the synthetic toy model along with a description of their functional form

**Triplet**	**O-information**	**Functional Description**
$\{S_1, S_4, D_1\}$	-0.230190	OR
$\{S_1, S_2, S_4\}$	-0.141707	XOR
$\{S_1, S_3, S_4\}$	-0.111444	Noisy XOR
$\{S_2, S_6, D_3\}$	-0.099158	Noisy XOR
$\{S_1, S_4, D_3\}$	-0.091215	Noisy XOR
$\{S_2, S_3, S_6\}$	-0.081516	XOR (broken)

$\{S_5, S_6, D_2\}$	-0.078728	OR

The table contains the four synergistic triplets specified when constructing the synthetic model, along with three other triplets.

Each "additional" (unspecified in model construction) triplet here as a variable with a strong pairwise mutual information with a variable from our specified synergistic triplets. It is therefore not surprising that these extra triplets appear. In our setup, they are akin to noisy XOR gates. For example, S_2 and S_3 are strongly related, so replacing S_2 by S_3in a synergistic triplet should retain a large amount of synergistic information.

Although constructed as an XOR gate, the synergy of $S_6 = XOR\ (S_2, S_3)$ is broken due to the dependence of S_3 on S_2. This increased the redundancy in the triplet, making the triplet less synergistic, decreasing its ranking. The synergy balance of the OR gate $D_2 = OR(S_5, S_6)$ was not as strong as the other OR gate. This is possibly related to the low entropy of variables S_5, S_6.

8.1 Possible Synergistic Triplets in the EASYcare data

The permutation test of O-information values, computed on triplets with a low dyadic mutual information (<0.05), found 184 triplets to be significantly synergistic, with p-values less than 0.05.

One such example, as mentioned in the main text is weight loss, dyspnoea, and locomotor pain, which all have weak dyadic associations but significant synergistic associations. Each pair of these signs has a weak pairwise association, giving a low sum of dyadic mutual information. When constrained to simple pairwise associations, we may (incorrectly) claim there is no association between these signs. However, due to their synergistic associations, knowing any two of these symptoms together tells us more about the third than if we only studied each pair of symptoms separately. This is seen by the triadic mutual information values in Table A1 row one, where $MI([Weight\ loss, pain\ locomotor], dyspnoea)$ and $MI([Weight\ loss, dyspnea], pain\ locomotor)$ are both greater than the sum of the corresponding pairwise associations.

The synergistic effect can be seen through the conditional probabilities of weight loss (w), pain locomotor (pl) and dyspnoea (d). The probability of weight loss is P(w)= 0.087. Only minor changes are seen when we condition on one of the variables;

$\mathrm{P(w|pl = 1) = \ 0.094, P(w|d = 1) = \ 0.084, P(w|pl = 0) = \ 0.077, P(w|d = 0) = \ 0.088.}$

However, conditioning on both variables results in a much larger change the probabilities;

$\mathrm{P(w|pl = 0, d = 0) = \ 0.048, P(w|pl = 0, d = 1) = \ 0.169, P(w|pl = 1, d = 0) = \ 0.126, P(w|pl = 1, d = 1) = \ 0.045}$. Supplementary Table 4 shows more possible synergistic triplets from the EASYcare dataset, with p-values <0.05 from a permutation test, without accounting for multiple comparisons. The sum of dyadic mutual information scores is given along with the corresponding triadic mutual information scores.

The sum of dyadic mutual information scores for a triplet (A,B,C) is given by

$MI(A, B) + MI(A, C) + MI(B, C).$

In many cases this is less than one or more higher-order mutual information values. These provide examples of when the information in all pairwise associations give less information than a higher-order association.

Supplementary Table 4 Showing some possible synergistic triplets from the EASYcare data. Synergy is clearly see when the sum of dyadic correlations is smaller than a higher-order correlation.

Synergistic Triplet	Sum of Dyadic Mutual Information	Triadic Mutual Information		
(A, B, C)		MI(AB→C)	MI(AC→B)	MI(BC→A)
Weight loss, Pain locomotor, dyspnea	0.0195	0.0369	0.0375	0.0188
Stiff restricted, UTI, CIRS-G Lower GI	0.0082	0.0263	0.0283	0.0244
Movement limitation, Stomachache, Mood complaints	0.0344	0.0493	0.029	0.0388
Weight loss, Fear of fall, CIRS-G Lower GI	0.0169	0.0275	0.0276	0.0239
Fear of fall, Vertigo, Other skin condition	0.016	0.0255	0.029	0.0206
Miction problems, UTI, Coughing	0.0058	0.0181	0.0199	0.016
Movement limitation, Hearing problem, CIRS-G Heart	0.0158	0.0288	0.0236	0.021
Urinary incontinence, other skin condition, CIRS-G ENT	0.0039	0.0163	0.0148	0.0175
Pain locomotor, Miction problems, CIRS-G Psychiatric illness	0.0169	0.0198	0.0241	0.0289
Vertigo, presyncope complaints, CIRS-G Neurologic	0.0071	0.0152	0.02	0.0177

Weight loss, sputum, CIRS-G Lower GI	0.005	0.0177	0.0134	0.0172
Presyncope complaints, UTI, Chest pain	0.0171	0.0295	0.0169	0.0261
Strength loss, Heartburn, Anxiety complaints	0.0119	0.0243	0.0182	0.0191
Fatigue, Presyncope complaints, CIRS-G Neurologic	0.0156	0.0201	0.023	0.026
Walk problem, Restricted action radius, Heartburn	0.1555	0.0134	0.1679	0.1672
Fall, Urinary incontinence, CIRS-G Upper GI	0.0219	0.0248	0.0222	0.033
Weight loss, Presyncope complaints, Heartburn	0.0028	0.0139	0.0146	0.0128
UTI, Heartburn, CIRS-G Endocrine and breast	0.0012	0.0128	0.0125	0.0128
Stiff restricted, other skin condition, CIRS-G Neurologic	0.0276	0.0359	0.0161	0.0383
Syncope, UTI, CIRS-G Vascular	0.0068	0.0175	0.0165	0.0144
Fear of fall, Visual problem, CIRS-G Psychiatric illness	0.0123	0.0144	0.0218	0.0232
Pain locomotor, Visual problem, CIRS-G Psychiatric illness	0.0158	0.0181	0.0215	0.0266
Strength loss, Change defecation, Hearing problem	0.0136	0.0132	0.024	0.0247
Fatigue, Weight loss, Heartburn	0.0074	0.0114	0.0187	0.0186
Weight loss, Fall, Heartburn	0.0034	0.0116	0.0146	0.0145

Urinary incontinence, Miction problems, CIRS-G Genitourinary	0.2168	0.2265	0.0748	0.1658
